# Merit overrules theory of mind when young children share resources with others

**DOI:** 10.1371/journal.pone.0227375

**Published:** 2020-01-03

**Authors:** James Stack, Carlos Romero-Rivas

**Affiliations:** 1 Department of Psychology, Liverpool Hope University, Liverpool, United Kingdom; 2 Department of Evolutive and Educational Psychology, Universidad Autónoma de Madrid, Madrid, Spain; Middlesex University, UNITED KINGDOM

## Abstract

Non-windfall approaches to sharing demonstrate pre-schoolers’ sensitivity to merit-based distributions of resources. However, such studies have not considered (1) whether epistemic aspects of task performance, such as the relative accuracy of a co-worker, influences pre-schoolers’ rates of sharing; and (2) how children’s emerging social understanding may impact resource allocations in high- and low-merit situations. These issues are of theoretical importance as they may provide new information about the scope of pre-schooler’s merit-based sharing behaviours. Moreover, as social understanding has been related to both increases and decreases in pre-schoolers’ levels of sharing, providing a merit-based assessment of this relationship would allow for a concurrent assessment of recent conflicting findings. In this study, three- and four-year-olds (N = 131) participated in an unexpected transfer task which was followed by a resource generation picture card naming task with a reliable or unreliable (high- or low-merit) co-worker (a hand puppet). The results showed that children engage in more generous rates of sharing with a high-merit co-worker. This suggests that merit-based sharing is apparent in young children and extends to epistemic aspects of task performance. However, such sharing was constrained by a self-serving bias. Finally, we were not able to detect an effect of children’s performance on the false belief task on sharing behaviours in the high- or low-merit trials, suggesting that these behaviours may not be modulated by social understanding during early childhood.

## Introduction

Sharing behaviours form part of a wider suite of prosocial skills which also include comforting, cooperation and helping [1]. As part of a recent trend to explore sharing behaviours at earlier developmental periods [2, 3, 4] attention has focused upon (a) factors motivating pre-schoolers’ decisions about whether to share (e.g., whether obtained sharing resources were jointly earned with a collaborative or high merit partner or merely freely received [5–9]) and (b) gaining insight into possible links between pre-schoolers emerging social understanding and their levels of sharing with others [10, 11].

Merit-based and collaborative studies collectively form the ‘non-windfall’ approach to sharing (where resources are earned). Although there are some notable exceptions [12, 13], such studies are framed within potentially costly first-person contexts, yet consistently demonstrate pre-schoolers capacity to share equally with others. This is in contrast with ‘windfall’ approaches (where resources are freely acquired). These studies demonstrate pre-schoolers desire to keep more resources for themselves in costly first-person situations [2, 3, 4] despite understanding the norms of fairness [14]. This suggests that non-windfall approaches offer an important route to help better understand prosocial behaviour at this key developmental period.

To date, assessment of co-worker contributions within pre-schooler non-windfall sharing studies has tended to focus on the more behavioural aspects of task performance (e.g., hard work and effort). Thus, we know little about whether pre-schoolers’ motivations to share with others extend beyond the more obvious and tangible aspects of co-worker contribution to also incorporate more cognitive and epistemic indices of successful task performance, such as reliability and relative accuracy of a co-worker [e.g., 15, 16]. Alongside these issues, to our knowledge there is no research that has concurrently assessed pre-schooler’s levels of social understanding and sharing behaviours in situations where the child’s motivation to share is influenced either positively or negatively by the contribution of a competent or incompetent co-worker. Therefore, the motivations behind the current study are twofold: (a) to assess whether pre-schoolers are able to use past accuracy of a high- or low-merit co-worker as a prompt to determine how much to share with a deserving or non-deserving co-worker; and (b) to determine whether pre-schooler’s levels of social understanding impact on sharing behaviours in high- and low-merit contexts. We reason that providing this assessment would contribute to our understanding of why and how pre-schoolers share with others.

### Non-windfall sharing and epistemic aspects of merit

In order to better understand the underlying issues of this study, it is necessary to first consider in more detail recent studies adopting ‘non-windfall’ dictator game methods. Such studies demonstrate that pre-schoolers who work alongside a collaborative [6–9] or high-merit co-worker [5,7] in order to jointly acquire resources tend to engage in more generous rates of sharing with their co-worker. In support of the merit-based approach, Kanngeisser and Warneken [5] demonstrated that pre-schoolers are sensitive to the relative work contributions of self and others. This was evidenced through varying the amount of resources they kept for themselves as a function of the amount of work performed by their co-worker.

Kanngiesser and Warneken’s findings sits readily alongside studies employing third person assessments of merit-based sharing. For example, Sloane, Baillargeon and Premack [17] demonstrated that infants preferentially attended to scenes when there was an unequal division of labour and yet an equal division of resources. Conversely, three-year-olds preferred an unequal division of resources to favour a story character who had finished a task when compared with a co-worker who lost interest and stopped before task completion [13].

Such findings provide an important contribution to our understanding of pre-schoolers’ sharing behaviours. However, it is important to look more closely at the way in which merit is operationalised in these studies. The key motivation within non-windfall studies is to create social situations that (a) mirror the collaborative foraging pressures felt within human evolution; and, therefore, (b) emphasise the importance of active participation as a means of acquiring resources. Emphasis here is placed on such attributes as the level of work input, productivity and effort [18, 19]. When framing merit as one of the three key principles for fairness, Chernyak and Blake [20] argue that ‘those who work harder should get more’ (p.1764).

There are, however, subtler ways in which relative merit can be assessed. Schäfer et al. [21] argue that, in Western society, determinants of relative merit are based on achievement as well as productivity. For example, two children studying for an exam may adopt the same work ethic (e.g., hard work and effort) in their attempts to obtain a desired outcome, yet additional cognitive attributes such as intelligence and memory will invariably result in one individual’s work being deemed to be of higher merit than the other. Thus, operationalising merit at this broader level allows for a meaningful assessment of how pre-schoolers reason about the relative merit of a co-worker’s competence in situations where epistemic aspects of task performance are key.

In support of this approach, a substantial body of research suggests that children preferentially respond to competent rather than incompetent actions. For example, infants [22] and pre-schoolers and older children [23, 24] selectively imitate competent, rather than incompetent models. Similarly, research from the selective trust paradigm demonstrates pre-schoolers preferences to learn novel words from, and demonstrate selective trust to, previously reliable informants [15, 16, 25]. This paradigm provides an ideal theoretical and empirical framework to assess merit-based sharing behaviours with pre-schoolers. In selective trust studies, children are familiarised with a reliable/unreliable adult informant who successfully/unsuccessfully attempts to label familiar items. For example, when presented with an every-day familiar item, such as a cup, the reliable informant would label this item accurately, whereas an ignorant informant would feign ignorance [15], or an unreliable informant would provide an inaccurate response (e.g., a dog) [16]. During test trials both informants are presented with an ambiguous item and provide two competing unfamiliar responses (e.g., by informants stating that it is either a ‘Wug’ or a ‘Dax’), and children are asked by the experimenter to indicate what they thought the item was called. Findings from these studies demonstrate that older pre-schoolers are sensitive to the past reliability of informants and will show a preference for the answers provided by the previously reliable informant. Moreover, these preschoolers made predictions about which informant would be accurate in similar future situations, prefer to endorse labels and direct questions to previously accurate informants. Such findings allow for an assessment of whether pre-schoolers will share more or less equally with a puppet co-worker whose productivity to help acquire resources (stickers) varies due to relative accuracy of responses rather than relative effort or hard work of each co-worker.

### Social understanding and merit-based sharing

Intuitively, it appears reasonable to assume that our ability to see the world through the eyes of others is related to a proclivity to share resources. In support of this assertion, recent studies using the ultimatum game methodology provide evidence suggesting that more advanced levels of social understanding, as measured through performance on various Theory of Mind (ToM) tasks, is related to more equal rates of sharing with others across early development. For example, Takagishi et al.’s [26] findings provide evidence of a link between five-year-olds’ false belief performance and greater rates of sharing when using a child-friendly version of the ultimatum game. Alongside these findings, measures of second-order false beliefs have also been linked to children’s increased rates of sharing when using the ultimatum game design [27].

In contrast, studies employing a dictator game approach demonstrate a lack of relationship [e.g., 27, 28] or only a partially supported relationship [29] between children’s ToM tasks performance and sharing behaviours. Importantly, Cowell et al.’s [10] recent findings provide evidence of an inverse relationship between social understanding and sharing, with pre-schoolers who passed a false belief task actually acting more selfishly than those children who failed this task when using the dictator game approach. Viewed collectively, the findings from dictator game studies question the assertion that ToM competence, acting as a mechanism allowing for greater perspective taking, produces more generous rates of sharing per se [e.g., 29].

These findings suggest that task demands within ultimatum and dictator game studies may uniquely impact young children’s rates of sharing with others. This is unsurprising as in typical dictator game studies a proposer is given an amount of resources (e.g., gummy bears or stickers) and is free to offer any amount of resources knowledgeable that there can be no retaliatory behaviours from the recipient. In contrast, in ultimatum games, any decision about how much to share is moderated by the awareness that a recipient can reject this offer leaving both proposer and recipient with nothing. This is problematic as any subsequent assessment of the relationship between social understanding and pre-schooler’s sharing will be determined by the specific strategies elicited by each approach [e.g., 27].

It is important to note that in one recent study with four- to six-year-olds, children with more advanced levels of social understanding (false belief) were observed making merit-based allocations within third-person stereotype-inconsistent contexts (e.g., where a girl works hard to make trucks) [12]. While informative, and suggestive of a link between social understanding and merit-based sharing, this study was based on a third-person assessment. Thus, this finding does not inform us of how pre-schoolers’ levels of social understanding would interact with their desire to share with a high-or low merit co-worker in costly first-person situations.

In view of the issues raised above, it may be fruitful to employ a novel approach that focuses less on (a) the consequences (or lack of) of resource allocation (as seen through differences between dictator and ultimatum game methodology); or (b) the wider motivations to share with others [e.g., 29–31]. Rather, the exclusive focus in the present study is on how ‘on task’ motivations, such as the co-workers’ level of competence, impacts 3- and 4-year-olds’ decisions about how to allocate earned resources with a high- or low-merit co-worker in first-person situations (where there is a potential cost to the child).

Importantly, our decision to focus on this age range was informed by prior research. As evidenced earlier, pre-schoolers at this age begin to demonstrate an understanding of the reasons why people share more or less equally with others [5–9] and also show sensitivity in their selective trust preferences for competent informants [15,16]. Finally, evidence suggests this as a key developmental period in the emergence of explicit false belief competence [32]. By adopting this approach, at this key developmental period, it is possible to test the full range of competing hypotheses in support of a positive, negative or null relationship between pre-schoolers’ emerging social understanding and their sharing behaviours.

### Predictions

The issues covered previously allow assessment of two important questions: how do children who pass or fail a standard false belief task respond in a dictator game situation (1) where resources have been acquired when working alongside a competent (high-merit) or incompetent (low-merit) co-worker?; and (2) where there are no consequences of retribution to the child proposer? There are a number of competing predictions here:

First, there will be greater rates of sharing from children who have acquired resources through working alongside a high-merit co-worker [5]. It is also predicted that children who have jointly acquired resources with a high merit co-worker, and who successfully pass a false belief task, will share more generously than children who have not passed this test [12]. The rationale here is that as such children can more readily represent another’s internal state, they should be able to appreciate that a high-merit co-worker would view a selfish offer as being unfair, having made an equal contribution to the acquisition of resources. This prediction would provide further support for the broader theoretical framework that views social understanding as a mechanism in promoting higher rates of sharing [29]. Conversely, children who have not passed the false belief task may be less able to make this inference and therefore offer a less generous amount to the deserving high-merit co-worker.

In contrast, it may also be the case that children who have passed the false belief task offer minimal resources to both a high- or low-merit co-worker. The rationale here is that such children may act more selfishly in a situation where they are: (a) less motivated to share with an undeserving, low-merit co-worker, and (b) are more able to infer that the recipient of a lower offer would be unable to engage in an act of reprisal in both high or low-merit situations. This prediction fits readily within the view that social understanding may offer children greater opportunities to act selfishly in situations where there is no cost to the individual [10]. It is also predicted that children who have worked alongside a low-merit co-worker and who have failed a false belief task would also keep the majority of available resources.

An alternative prediction here is that there will be no relationship between false belief task performance and pre-schoolers sharing behaviours [27, 28] in high or low merit conditions. Such outcomes would be consistent with the view that pre-schoolers are as yet unable to use fully meta-representational forms of social understanding in order to guide merit-based sharing behaviours with deserving or non-deserving co-workers. Following this line of reasoning a final prediction states that pre-schoolers sharing behaviours will differ solely due to the relative merit of each co-worker.

## Materials and methods

### Participants

A total of 131 three- and four-year-olds (73 boys and 58 girls) participated in the study. There were 76 three-year-olds (*M* = 3.56 years; *SD* = 0.27 months) and 55 four-year-olds (*M* = 4.37 years; *SD* = 0.28 months). All children were recruited from three nurseries based in the North of England. Parents of participants and each child gave written consent in advance.

The research was approved by the Liverpool Hope University Research Ethics Committee.

### Materials and procedure

#### Social understanding

A variation of the standard unexpected transfer task was used [33]. Children were introduced to a glove-puppet crocodile (Colin) who liked to eat lemons. He hid the lemon in one of two boxes (blue or red) before exiting the scene. In his absence the experimenter moved the lemon from its’ original location to an empty box. Colin then re-appeared and each child was asked '‘where will Colin look for the lemon?’ (belief question). In order to determine that children understood this task, each was also asked ‘and where is the lemon now?’ (control question). All children who were included in the main analysis correctly answered this question.

#### Merit-based sharing

After the social understanding task, children were asked whether they would like to play a game with a glove-puppet (either Tommy the Tiger or Polly the Parrot; the choice of puppet was based on the child’s preference) to see if they could get stickers. In both puppets there was a small mechanism that squeaked each time it was squeezed. This was designed to articulate a response to the experimenter that was then deciphered to the child as being accurate or inaccurate. Puppets were used in this study as it allowed the researcher to control the relative contributions of the high- and low-merit co-worker within each condition [5, 6]. Children were then presented with a small plastic tray that contained a choice of 4 different coloured stickers (red, blue, green or yellow). Each child was asked what their favourite coloured sticker was. Upon choosing (e.g., red) they were told that this was also the puppets’ favourite coloured sticker. These stickers were taken off the tray and placed on the table in-between the child and the puppet (who remained in attendance) with the non-chosen stickers removed from view. Children were presented with a wooden tray that held 12 picture cards, placed in two rows of six. Children were told that they would have a practice round to see if they could name each picture card and that once this task was completed they would engage in the real game to get the stickers.

The experimenter then pointed to each of the 12 picture cards in turn and the child was asked to name it. The images presented on each card were: (1) a flower, (2) a ball, (3) a cat, (4) a tree, (5) a lion, (6) a zebra, (7) a drum, (8) balloons, (9) a dog, (10) a shoe, (11) a bird, and (12) a rabbit. Children typically got all 12 items correct at the first time of asking. There were occasions where children named the shoe a trainer, the zebra a horse or the bird a chick. These answers were equally valid and so were treated as being technically correct. On occasions where children did not know the name of an item the experimenter told them what it was and upon completion of their answers were asked if they could recall it. Children were deemed to be successful and the experiment progressed only upon correctly naming all picture cards during the practice phase. The rationale behind this choice of merit task was to utilise and modify methodology from the selective trust paradigm [15, 16] in order to assess whether children could move beyond the more basic indices of merit (e.g., hard work) and modify sharing behaviours based on epistemic aspects of task performance.

Children were informed that the puppet co-worker, who was sitting nearby, had been watching while they accurately named the picture cards. Evidence demonstrates that infants and younger pre-schoolers use lower level forms of social understanding to correctly infer that presence (or absence) leads to knowledge (or ignorance) of changes within the environment [34, 35]. Moreover, previous research shows that pre-schoolers can also match observable reality (e.g., what a puppet has seen) with subsequent knowledge claims [36]. Thus, pre-schoolers within the present study are developmentally capable of making the inference that as the puppet was in attendance during the practice round, it should be able to accurately name each picture card item when prompted by the experimenter. Each child was then informed that they would play the game again, but this time for real, in order to get stickers. The same procedure was repeated, with each child successfully naming the first six picture cards. After each successful naming of each picture card, a sticker was placed immediately underneath it. Therefore, upon completion of this phase all children had accurately recalled half of the items and therefore contributed 50% of the stickers to the pot of resources to be shared later in the procedure. Children were also informed that it was now the puppet’s turn to see if it could also contribute to the pot of stickers. Each child observed as the experimenter asked the puppet to name the six remaining picture cards. The puppet responded to each question by turning to face the experimenter and making a high-pitched squeaking noise.

#### High merit

In the high merit condition, the puppet, when prompted, successfully named each of the remaining six picture cards. Therefore, the puppet had demonstrated competence in accurately recalling the names of the picture cards and in turn had successfully earned the remaining 50% of available stickers. These were then added to the original pot resulting in a combined total of 12 stickers (six earned by the child and six earned by the puppet).

#### Low merit

In the low merit condition, the child again accurately named the first six picture cards (and earned six of the available twelve stickers). However, in contrast to the high merit condition, the puppet failed to accurately name any of the remaining six picture cards. For example, when the experimenter pointed to the picture of the dog the puppet intimated that it was something else (e.g., a flower). Each time the puppet failed to correctly name the picture card the experimenter said ‘that’s not a *** that’s a ***…. That was wrong… No sticker!’. The sticker was placed face down on the far side of the table. Therefore, at the end of this phase the child was able to reason that the puppet had failed to label any of the items correctly and had made no contribution to the pot of available stickers. Each child was then verbally informed of this outcome but told that the stickers could still be earned if they were able to correctly name the remaining six picture cards. Children were asked to name each of the remaining six items and were rewarded with a sticker being placed underneath each picture card for each accurate response. These were then added to the original pot resulting in a combined total of 12 stickers (twelve earned by the child and none earned by the puppet).

#### The sharing task

Having worked alongside either a high- or low-merit puppet in order to generate a pot of twelve stickers children observed as the experimenter counted out aloud as he placed the stickers on a smaller tray into two rows of six. The puppet was placed approximately fifty cm away from the child. A tray was placed in front of the puppet and a second tray placed in front of the child. Children were then informed that they were to be given the stickers and that they could keep them all, give them all away or share them with the puppet. In order to reduce any additional levels of social pressure, the experimenter told the child that he was going to be busy for a moment while they decided what to do. During the allocation phase, the experimenter turned to one side, picked up a book and began reading it. Upon completion of this task, the number of stickers shared was noted. Children were finally informed that they had done very well playing the game and because of this they could have some extra stickers to take home.

#### Data coding and analysis

We analysed the data using a factorial ANOVA, with *age* (3, 4 years), *gender* (boy, girl), performance on the *false belief* task (pass, fail), and puppets’ *merit* (high, low) as between-subjects variables, and the number of stickers kept by children as dependent variable. Moreover, in order to explore whether demographic factors modulated the results of the social understanding task, we carried out an additional factorial ANOVA with *age* and *gender* as independent variables, and performance in the *theory of mind* task as dependent variable.

## Results

### Analysis of the number of stickers kept by children

**Fig 1:**
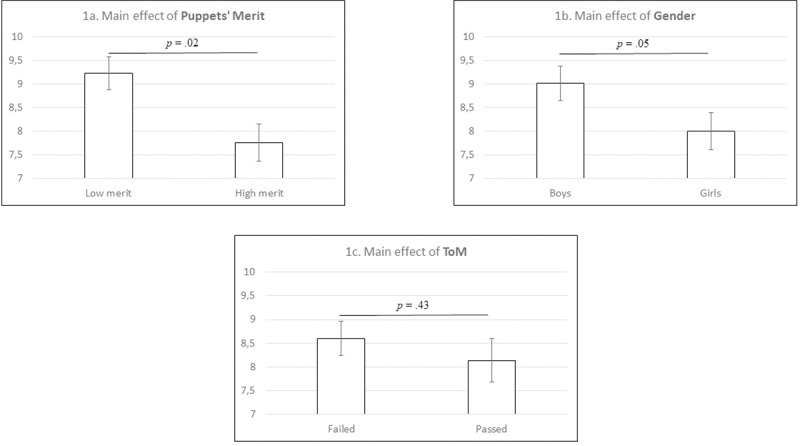
Amount of stickers kept by children depending on Puppet’s Merit (1a), Gender of children (1b), and outcome in the ToM task (1c). Error bars represent standard errors.

In this analysis, Levene’s test for equality of variances was not significant, *W*(15,115) = 1,68, *p* = .07. The results of the factorial ANOVA showed a significant main effect of *merit*, *F*(1,115) = 5.23, *MSE* = 9.23, *p* = .02, *ηp*^*2*^ = .044. Children who interacted with the low-merit puppet kept a higher amount of stickers (*M* = 9.22, *SE* = 0.35) than those interacting with the high-merit puppet (*M* = 7.76, *SE* = 0.39; **Fig 1A**). In addition, we observed a marginally non-significant main effect of *gender*, *F*(1,115) = 3.84, *MSE* = 9.23, *p* = .05, *ηp*^*2*^ = .032, with boys keeping a higher number of stickers (*M* = 9.01, *SE* = 0.36) than girls (*M* = 8.00, *SE* = 0.39; **Fig 1B**). All other main effects and interactions between variables were not significant (all *p* values > .22; **Table 1**).

**Table 1:** Descriptive statistics for the analysis of the number of stickers kept by children.

Age	Gender	ToM test	Merit	Mean	SE
Three	Boy	Fail	High	8.29	.81
			Low	9.07	.78
		Pass	High	8.60	1.36
			Low	9.22	1.01
	Girl	Fail	High	7.80	.96
			Low	9.75	.88
		Pass	High	7.50	1.24
			Low	8.40	1.36
Four	Boy	Fail	High	8.87	1.07
			Low	9.75	1.07
		Pass	High	8.00	1.75
			Low	9.73	.92
	Girl	Fail	High	6.40	1.36
			Low	8.87	1.07
		Pass	High	6.12	1.07
			Low	7.50	1.52

Regardless of the main effect of *merit*, our results also showed that children had a self-serving bias [5]. Therefore, even though children were able to consider different contributions children who interacted with the low-merit puppet (*t*(71) = 9.31, *p* < .001, *d* = 2.21) and those interacting with the high-merit puppet (*t*(58) = 4.51, *p* < .001, *d* = 1.18) kept more stickers for themselves compared to an equal allocation (i.e., sharing/keeping 6 stickers).

Despite not finding a significant effect of the performance on the *false belief* task in the analysis, a Sobel test was conducted to explore whether ToM had a mediational role in a model where puppets’ *merit* was the independent variable, and the amount of stickers kept by children the dependent variable. The result of the test (*z* = -0.33, *p* = .74) pointed out that ToM did not have a mediational role in this model.

Finally, and in order to better understand the nature of the non-significant effect of ToM in the main analysis, we conducted an equivalence testing, following the TOST procedure [37]. In this analysis, we explored whether we could reject the hypothesis that the difference in sharing behaviour between children who passed and not passed the ToM task was less extreme than an equivalence bound (which would mean that the number of stickers kept by both groups would be equivalent). The equivalence bound was established after considering the effect size of a non-significant main effect of ToM in sharing behaviour in children reported in a previous study (*w* = .03) [31]. Taking into account that these researchers used 145 participants, a two-sided test with an alpha of .05 would have 33% power to detect a critical *χ*^*2*^ = 5.66 (*w* = .20); importantly, this effect size falls between what is usually considered a small (*w* = .10) and a medium effect size (*w* = .30), thus being somewhat similar to a *d* = .35. Once established the smallest effect size of interest for determining the equivalence bound, we run the test, finding that it was non-significant, *t*(113.54) = -1.14, *p* = .13. Since we did find evidence to reject the alternative hypothesis, the test suggests that the number of stickers kept by children who passed the ToM task and children who did not were not equivalent (**Fig 1C**).

### Analysis of the effect of demographic factors on the performance in the ToM task

Levene’s test for equality of variances was not significant in this analysis, *W*(3,127) = 2,20, *p* = .09. The factorial ANOVA did not provide significant results (all *p* values > .10). Thus, in the three-years-old group 25 of 76 children tested passed the false belief task (*M* = 0,32, *SD* = 0,47), while in the four-years-old group 26 of the 55 children tested passed the task (*M* = 0,47, *SD* = 0,50). Furthermore, 28 boys from the 73 (*M* = 0,38, *SD* = 0,49) and 23 of the 58 girls (*M* = 0,40, *SD* = 0,49) passed the ToM task. These data are consistent with trends reported in earlier studies in this area [e.g., 25, 27].

## Discussion

The current study assessed (a) whether pre-schoolers use epistemic indicators of relative merit as a basis for subsequent sharing behaviours with others; and (b) whether levels of social understanding differentially influence rates of sharing in high and low merit situations. Our results provide further support for the view that pre-schoolers are sensitive to the relative merit of a co-worker and allocate resources accordingly [5].

Importantly, one key difference between this and other studies assessing merit-based sharing with pre-schoolers rests on the move away from assessment of merit based on such attributes as hard work or effort [18,19], to an assessment of epistemic aspects of merit based on relative accuracy of the co-worker [15,16]. Hence, it is important to highlight how our findings relate to the results observed within the selective trust literature. Findings from studies within this paradigm show pre-schoolers preferences for an accurate informant’s testimony extending beyond the informants current domain of knowledge. Thus, pre-schoolers view accurate informants as also being more competent in unrelated domains (e.g., naming object functions), and they are also more likely to seek help from such sources [15, 16]. This contrasts with research suggesting that pre-schoolers preferences are based on simple heuristics (e.g., they prefer to learn new words from strong or attractive models, as opposed to from a model that is weak or unattractive) [38, 39]. Our findings suggest that pre-schoolers not only have the social cognitive capacity to selectively prefer accurate over inaccurate informants [16], but are able to use co-workers past levels of accuracy as the basis from which to make equitable sharing decisions with deserving or non-deserving co-workers. While this finding sits readily alongside richer, more sophisticated accounts of selective trust [38], it must also be noted that no additional measures beyond sharing behaviour were obtained from our study. Thus, while pre-schoolers sharing behaviour appears to preferentially favour high-merit co-workers, further research is needed in order to concurrently assess whether sharing preferences would be manifested in a more general inclination to trust this source across, and beyond the domains considered above.

Alongside these issues, our findings raise important theoretical questions related to why pre-schoolers resource allocation rates differed with high- and low-merit co-workers. Before considering the richer merit-based account, it is important to note here that our data could also be interpreted as providing support for either a lean associationist or a reputational management account [40, 41, 42]. Regarding the former, it may be argued that pre-schoolers within this study simply formed a more positive association with the puppet that was linked to the conferring of the stickers. However, our findings are more persuasively explained through a richer merit-based interpretation. For example, if we follow the ‘positive association’ line of argument, we would expect to find empirical support showing that children give more to recipients who are in possession of (or at least who are associated with) resources (as in our high merit condition), compared with those who are not (as in our low merit condition). Recent evidence is diametrically opposed to this view. Different studies demonstrate that preschoolers have a preference to allocate more resources (between other people) and share at greater levels (between self and others) with resource-poor, rather than resource-rich recipients [43, 44, 45]. Alongside these behavioural findings, research also suggests that older preschoolers have normative expectations for more charitable distributions to poor, rather than rich recipients [46]. Thus, any preference to share more with the puppet that was awarded with stickers is unlikely to be influenced by an associationist strategy, but rather reflects the levels of competence demonstrated by the high merit puppet co-worker.

Due to the co-worker puppet remaining present during the sharing task, it could also be argued that any tendency to share more with a high merit puppet may to some degree also be due to pre-schoolers desire to manage their reputation [42]. Such reasoning (e.g., thinking about what the puppet would think about them) involves more advanced forms of social understanding (e.g., second order false belief), and is considered to be beyond the capabilities of pre-schoolers [47]. However, pre-schoolers are sensitive to social cues (e.g., the presence of watching eyes) and modify their levels of generosity in such contexts [44, 45, 46]. This suggests that three- and four-year-olds within the present study, at least implicitly, are able to make inferences about how their co-worker puppet would view their sharing behaviour. While this is an interesting interpretation offering new avenues for further research, it must be noted that in Kanngeisser and Warneken ‘s (2012) merit-based study [5], the co-worker puppet left the room at the critical moment when the child shared the earned resources, yet pre-schoolers still allocated more resources to a high-merit co-worker. This would suggest that while reputation management may be important in iterative merit-based exchanges between co-workers, pre-schoolers in the one-shot dictator game approach within the present study again appear to be basing their sharing decisions on their co-workers relative merit.

Moving beyond these accounts, our findings show some similarity with research that has assessed pre-schoolers’ sensitivity to the effect of free-riding. For example, 3-year-olds share less with others in situations where a potential co-worker has chosen not to collaborate with the child, but to do something else, during their attempts to obtain resources [6]. Moreover, Yang et al. [48] demonstrated that 4- to 5-year-olds hold negative evaluations and punish free-riders within both first- and third-party (where there are no direct consequences for the child) contexts, suggesting that young children are also morally conscious of the normative expectations which underpin collaborative exchanges.

Are pre-schoolers in the present study using a similar rule? For example, our pre-schoolers were: (a) aware that puppet had remained present during the practice round of the procedure (where the child correctly labelled each picture card) and therefore (b) should know the name of each image [34, 35]. Subsequently, the co-worker should be capable of using such knowledge to earn resources by accurately informing the experimenter of the name of each picture item when prompted. In contrast to these expectations, children who worked alongside the low-merit puppet observed an inaccurate and incompetent informant and thus had to rectify each incorrect answer by providing the experimenter with the correct labels for each picture card. This would suggest that pre-schoolers were viewing the lack of contribution in the low-merit trials in a similarly negative light to children assessed in the free-rider studies cited previously [6, 48].

It should also be noted that by punishing the non-contributing low-merit co-worker and rewarding the high-merit co-worker with a more generous allocation, pre-schoolers may be engaging in evolutionary developed behaviours designed to promote the probability of engaging in behaviours with reciprocal positive outcomes (e.g., facilitating engagement in future activities with a high-merit co-worker) [49, 50]. This line of reasoning, especially when considered alongside the possibility of pre-schoolers having reputational concerns (see above) opens up the possibility of exciting new areas of research.

Despite the fact that children (even in the low-merit condition) on average shared a significant amount of resources with their co-worker, it is important to note that children in our study also demonstrated a self-serving bias [5]. Here, our findings provide an interesting comparison with windfall assessments of pre-school sharing. Evidence from studies using the windfall approach demonstrate pre-schoolers awareness of the norms of equal sharing with others [14], yet such children unfairly allocate more resources to themselves in costly first-person situations [2–4, 14]. Data from our study are only partially consistent with these findings in that pre-schoolers sharing behaviour was constrained by a self-serving bias (keeping more than half for themselves) in both high- and low merit conditions. We interpret this as highlighting the utility of non-windfall approaches [5–9] in supporting pre-schoolers in their shift toward other-oriented forms of sharing.

In addition, the present study was also motivated to help better understand the current divergence of findings by assessing whether resource allocations with a high- or low-merit co-worker would be additionally influenced by the child’s current level of social understanding. By providing pre-schoolers with either a high- or low-merit co-worker, we were able to assess whether children who passed the false belief task would be motivated to act in either a more other-regarding [29] or self-serving manner [10]. The final alternative here was that despite providing this motivation context, pre-schoolers would not yet have the capacity to use social understanding as a basis for meritocratic sharing decisions [27, 28]. The current findings offer no support for the more prosocial or the more self-serving interpretations.

Rather, our findings add to existing research showing no relationship between ToM and sharing preferences [27, 28]. Liu et al. [28] reason that this lack of relationship, and the more general divergence between findings from dictator game and ultimatum game approaches which have shown support for this relationship [26, 27], may be due to the less strategic nature of task demands within dictator game tasks. However, as children in the present study experienced explicit non-windfall cues (whether or not a co-worker had earned resources), this further suggests that performance on measures of social understanding may be less closely related to pre-schoolers sharing behaviours in dictator games than originally thought. Therefore, our findings appear more consistent with Imuta et al.’s [51] recent argument, which is distanced from the social understanding account and rather frames sharing more as a social convention which children acquire through routinely reinforced parental instruction.

Nevertheless, the non-significant effect of ToM on children’s sharing behaviours should be treated with caution, as the test for equivalence indicated that the amount of stickers kept by children who passed or not passed the false belief task were not equivalent. This means that we cannot reject the hypothesis that the effect of ToM on sharing behaviours is at least as extreme as a medium effect size (given the equivalence bound we established in our analysis). In any case, and given that we did not find a significant effect of ToM in the main analysis, or a mediational role of ToM in a model where puppets’ merit was the independent variable and the amount of stickers kept by children the dependent variable, our results still appear consistent with the literature distancing social understanding from sharing behaviour [51].

On a side note, we obtained a marginally non-significant effect of gender within our findings. Although the effect of gender on sharing behaviour is mixed, with some studies showing no effect [2,14], numerous studies have demonstrated a gender difference in sharing behaviours with boys sharing less than girls [52, 53, 54]. It is worthwhile noting here that these findings reflect a tendency for boys to engage in more selfishly motivated behaviours, especially at earlier developmental periods, with this effect disappearing in later childhood [54]. Moreover, boys appear as more competitively motivated than girls [53], and are less sympathetic to another’s situation when sharing valued resources [54]. Thus, these findings may go part-way in explaining why boys in the present study were less motivated to share, even when working alongside a high-merit co-worker.

### Limitations

It is important to call out some caveats in our study’s design. First, the social understanding task we used (a variation of the standard unexpected transfer task [33]) only allows for dichotomous responses (either correct or false answers). Although this task has been widely used to study social understanding in pre-schoolers [32], we believe that a different task/measure contributing continuous responses would allow researchers to obtain a wider variability of responses, thus avoiding undesirable effects (such as guessing). Additionally, and as mentioned above, our analyses showed that the effect of ToM on children’s sharing behaviour was not statistically significant; but, at the same time, the equivalence test pointed out that the amount of stickers kept by children who passed the social understanding task and those who did not were not equivalent. Hence, future studies may increase the statistical power and/or pre-establish effect sizes of interest to determine whether ToM has a mediational role in pre-schoolers sharing behaviours.

## Conclusion

Our findings suggest that children as young as three and four-years-old are able to use the reliability of co-workers as a cue for merit-based sharing behaviours. While the developmental period between three and four years of age has been extensively considered to be a key developmental period in false belief understanding [32] our results do not allow us to conclude whether false belief performance is a reliable predictor for sharing behaviours at this early age. Consequently, this warrants the need for follow-up studies that provide broader developmental measures of social understanding during and beyond the pre-school period [e.g., 55, 56]. These findings also raise a number of further questions: Why is it that pre-schoolers appear to persist with a self-serving bias even in instances where they have earned resources alongside a high-merit co-worker? It may well be that this behaviour becomes less pronounced across childhood [14]. Future research may also explore the scope of merit-based sharing. For example, are pre-schoolers expectations of high- and low-merit performance framed within the here and now, or do they act more generously with people they may rely on in future situations? If so, this would suggest that pre-schoolers (a) show sensitivity in their attempt to manage reputation; and (b) engage in directly reciprocal merit-based exchanges. Finally, would children expect a high merit co-worker to demonstrate competence across unrelated domains? [14, 15] Again, the points raised here suggest the need for further developmental research across the pre-school period and beyond.

## Supporting information

S1 Data (CSV)
